# Long-Term Effect of Guided Implant Surgery on Clinical Outcomes and Peri-Implantitis of Maxillary Implants—An Observational Cohort Study

**DOI:** 10.3390/jcm12134432

**Published:** 2023-06-30

**Authors:** Emitis Natali Naeini, Hugo De Bruyn, Ewald M. Bronkhorst, Jan D’haese

**Affiliations:** 1 Department of Dentistry, Radboud University Medical Centre, 6525 GA Nijmegen, The Netherlands; 2 Department of Periodontology and Oral Implantology, Faculty of Medicine and Health Sciences, University of Ghent, 9000 Gent, Belgium

**Keywords:** guided surgery, computer-assisted implant placement, flapless surgery, long-term, peri-implantitis, technical complications, biological complications

## Abstract

(1) Although the accuracy of static computer-aided implant surgery (sCAIP) is well reported, information on its long-term effect on peri-implant health and complications is scarce. (2) Twenty-six patients initially treated were recalled. Implant survival, radiographic bone level, peri-implant health, and complications were registered. A multilevel regression model was applied to study the relationship between the research variables. (3) Sixteen patients participated in this study (average age 58.5 years; range 27.8–73.8). The mean follow-up time was 9.1 years (range 7.3–11.3). Two implants failed, resulting in a survival rate of 97.1%. The mean bone level change corresponded to a loss of 0.63 mm (SD 1.90) for the whole group, 0.17 mm (SD 1.46), and 0.91 mm (SD 2.09) for tooth- and mucosa-supported guides, respectively. The mean PPD for the total group was 4.24 mm (SD 1.25), and 3.79 mm (SD 0.97) and 4.51 mm (SD 1.33) for the tooth- and mucosa-supported guides, respectively. Four implants (6.3%) were diagnosed with peri-implantitis. Coronal deviation was slightly associated with having a negative impact on bone level at follow-up, but this was not statistically significant. Seven patients (43.8%) experienced technical complications. Biological complications were seen in 3/16 patients (18.75%). (4) SCAIP may contribute to more predictable implant placement; the long-term clinical outcome is similar to conventional nonguided surgery.

## 1. Introduction

Dental implant surgery involves the placement of an implant into the jawbone, either by conventional open flap surgery, free-handed flapless surgery, or computer-aided implant surgery.

The advantages of flapless surgery include less postoperative bleeding, reduced swelling, less pain, and rapid postsurgical healing [1,2,3]. Flapless surgery is also less time-consuming [4]; reduces the risk of marginal bone loss due to avoiding raising a flap, and facilitates soft tissue management during implant surgery [5]. However, flapless surgery requires careful planning to ensure precise placement and predictable results. The main disadvantage of free-handed flapless implantation is the inability to properly assess the final location of the implant in relation to the volume or structure of the underlying bone. This increases the risk of bone perforation [6], which could cause major aesthetic problems and even implant loss [7]. Computer-aided implant surgery (CAIP) has emerged as a promising technique that utilizes cone-beam computed tomography and computer-aided design/computer-aided manufacturing (CAD/CAM) technology to plan and place dental implants in a predetermined position and orientation according to restorative goals and anatomic limitations in order to overcome the challenges associated with free-handed flapless surgery [8,9].

CAIP is classified based on the level of guidance (partially vs. fully) and the possibility to adapt the surgery intraoperatively (static vs. dynamic). Static computer-aided implant placement (sCAIP) is a fully guided approach that involves restrictive osteotomy preparation as well as implant placement through a prosthetically driven stereolithographic surgical guide [10,11]. Whereas dynamic computer-aided implant placement (dCAIP) reproduces the virtual implant position straight from computerized tomographic data, allowing intra-operative changes [10].

Several studies have evaluated the long-term survival of implants placed using sCAIP. A systematic review and meta-analysis of 10 RCTs, conducted by Tattan et al. (2020), reported >98% survival after 12 months post-loading [10]. Another systematic review of 13 studies with a total of 2019 implants showed a 97% survival over a mean follow-up period of 22.6 months [12]. The literature describes sCAIP as a reliable technique, however, clinicians should take account of the safety margins needed with guided surgery [12].

One of the chief factors in the long-term success of dental implants is the preservation of stable peri-implant bone levels. Moraschini et al. (2015) [12] reported a marginal bone loss of 1.45 mm for sCAIP during a 1–4-year follow-up period. One RCT reported a mean marginal bone loss of 0.7 ± 1.3 mm, 3 years after loading [13]. Despite the significance of long-term prognosis outcomes when evaluating flapless sCAIP, not many studies report on bone level changes over a long period of time. However, the results available suggest that stable bone levels can be achieved over long-term follow-up periods [12].

Another important criterion to evaluate success is the long-term incidence of peri-implantitis. According to a consensus report published in 2017 [14], the diagnosis of peri-implantitis involves the presence of bleeding and/or suppuration, increased probing depths as compared to previous examinations, as well as ongoing crestal bone loss beyond regular initial bone remodelling [14]. When previous examination data is unavailable, peri-implantitis is defined by probing depths > 6 mm, bone levels > 3 mm apical to the most coronal part of the intraosseous aspect of the implant, combined with the presence of bleeding and/or suppuration [14]. The prevalence of peri-implantitis varies considerably among studies due to inconsistent definitions, different reporting methods, and study characteristics [15]. A systematic review of 2019 implants placed using sCAIP reported that the prevalence of peri-implantitis was 14% after a follow-up time of 1–4 years [12]. Another retrospective multi-centre study reported only a 1.7% prevalence of peri-implantitis after a 10-year follow-up period [16].

When evaluating implant success, the occurrence of biological and technical complications is highlighted more often. Biological complications include peri-implant pathology, including mucositis and peri-implantitis [17], marginal fistula [17], and implant fenestration [9,12]. Technical complications include surgical template fractures [10], misfit of the surgical template during surgery [10], prosthesis fractures [17], loosening of retaining screws [17], and discrepancies between abutments and implants [17]. One study reported 7.1% technical (minor template-related) complications and 1.7% biological complications (peri-implantitis) over 10 years [16]. A systematic review and meta-analysis reported a cumulative survival rate of 83.9% [18] to 100% of the prosthesis after 1–4-years [12]. It is not surprising that accuracy of implant placement is the most commonly reported outcome measure of CAIP [10]. Systematic reviews show that sCAIP yields higher accuracy in all domains as compared to free-handed and partially guided implant placement [9,10,11,17,19,20]. Mucosa-supported and tooth-supported guides also performed statistically significantly better than bone-supported guides. Even though the accuracy of computer-assisted implant placement is well reported, to our knowledge, there are no studies correlating the effect of higher precision during placement to the beneficial clinical outcome of these implants in the long term.

## 2. Aim

The aim of this study is to analyze the long-term effect of guided implant surgery on peri-implant health by using peri-implant bone level and probing pocket depths as the parameters and complications.

A secondary aim is to analyze the effect of the accuracy of implant placement on the peri-implant bone level and pocket depth in the long-term.

## 3. Material and Methods

### 3.1. Study Design

This is an observational cohort study.

### 3.2. Setting

Originally, 26 cases exhibiting a partially (*n* = 13) or fully edentulous (*n* = 13) maxilla were chosen for implant treatment by means of the Facilitate software system (Astra Tech) for virtual treatment planning as well as flapless implant placement. The patients were all referred and treated by one surgeon (JD). Patients were excluded when there was insufficient bone available for safe implant placement and if they were undergoing Bisphosphonate treatment or had undergone head and neck radiotherapy. Smokers were not excluded.

All patients were periodontally examined at intake and treated when necessary. Hopeless teeth were extracted at least 3 months before implant surgery. A provisional immediate removable denture was delivered to the patients. A diagnostic case was fabricated using an irreversible hydrocolloid (Cavex CA37, fast set, Cavex Holland BV, Haarlem, The Netherlands). These master casts were mounted into an articulator, after which a prosthetic set-up was fabricated. According to this wax-up, a prosthesis was made comprising small radiographic glass spheres that were embedded in the resin of the prosthesis. The glass spheres acted as radiographic markers, allowing the temporary prostheses to also be used as scanning templates.

### 3.3. Planning Procedure

CT-scanning was performed with the prosthesis in situ using a Siemens Somatom Definition 64-slice dual source CT-scan. This was conducted in accordance with the dual scan procedure defined in the protocol by Materialise (Materialise N.V., Leuven, Belgium). Subsequently, a second CT scan (dual scan) was taken of only the prosthesis. These CT images were transformed into DICOM images (Digital Imaging and Communications in Medicine) and converted into a 3D virtual model using the Facilitate™ software system (Astra Tech AB, Mölndal, Sweden). It was ensured that the implant locations, as well as the implant lengths and widths, were deliberated in a prosthetically driven way. Using a stereolithographic machine, layers of liquid polymer were laser-cured to fabricate a surgical guide.

### 3.4. Surgical and Prosthetic Procedures

Surgery was performed under local-regional anaesthesia, with appropriate aseptic and sterile conditions. Before starting the intervention, the surgical guide was disinfected for 15 min using a 0.2% chlorhexidine solution. The guide was placed on top of the mucosa (a mucosa- supported guide) or on top of the residual teeth (a tooth-supported guide) and fixed using osteosynthetic screws. The osteotomies were prepared at 1500 rpm under copious irrigation and limited to the desired depth by means of a vertical stop on the drills. Gingival punching was not performed prior to implant site preparation. Either 2, 3, 4, or 6 OsseoSpeed™ implants (Astra Tech AB, Mölndal, Sweden), which had a TiO_2_-blasted fluoride-modified surface, were implanted into the maxilla with a maximum torque of 50 Ncm. Subsequently, 20° UniAbutment or angulated abutments (Astra Tech AB, Mölndal, Sweden) were placed onto the implants and torqued to 15 Ncm. Height and angulation were determined prior to surgery using the planning software package. After mounting the pick-up copings, an abutment level impression was made with silicone material (Permadyne Penta H, ESPE, USA). This was performed by using the existing removable prosthesis as a tray. Within 8 h, a temporary screw-retained, fibre-reinforced acrylic bridge was given to the patient and fitted in the mouth by connecting it to the abutments. Occlusion and articulation were corrected where necessary. No cantilevers were present in the temporary bridges so as to avoid excessive occlusal and non-axial forces. Postoperatively, each patient was prescribed clindamycine (300 mg, 3×/d), ibuprofen (600 mg), and chlorhexidine rinse. After 48 h, a postoperative visit was planned to check and adjust occlusion and articulation. The final prosthetic construction was performed at least 3 months after implant installation by the referring dentist, being either a screw-retained metal-ceramic fixed prosthesis or a metal-resin fixed prosthesis.

### 3.5. Accuracy Analysis

Around four weeks postoperatively, a new CT scan was taken. Software (Mimics 9.0, Materialise N.V.) was used to fuse the images of the placed implants over the planned implants. The locations and axes were then compared. An object registration was carried out in order to evaluate the deviations between the planned and the placed implants. This allowed the software to pair-wise align the pre-operative 3D images of the jaws with their postoperative counterparts. The jaws were matched using an iterative closest point (ICP) algorithm. The established coordinate transformation operations could be applied to the 3D representations of the planned implants, thereby allowing relative comparisons of the postoperative implant positions. Four deviation parameters were defined and calculated: global, angular, depth, and lateral deviation. This was performed by using the coordinates of their respective coronal and apical points. Global deviation was described as the 3D distance between the coronal (or apical) centres of the corresponding planned and placed implants. The angular deviation was calculated by measuring the 3D angle between the longitudinal axes of the planned and placed implant. When calculating the lateral deviation, a reference plane was first defined by drawing a perpendicular plane to the longitudinal axis of the planned implant, going through its coronal (or apical) centre. The lateral deviation was then calculated by measuring the distance between the coronal (or apical) centre of the planned implant and the intersection of the longitudinal axis of the implant with the aforementioned reference plane. Depth deviation was measured using a plane parallel to the reference plane, which goes through the coronal (or apical) centre of the placed implant, and then measuring the distance between the coronal (or apical) centre of the planned implant and the intersecting point of the longitudinal axis of the planned implant. The results of the accuracy analysis [21] and clinical outcomes at 1-year follow-up [22] have been published by D’haese et al. (2012, 2013).

### 3.6. Participants

The original study group was recalled 7–11 years after implant placement for a research examination by an external, independent researcher (ENN) from Ghent University, Belgium. Inclusion and exclusion criteria were predetermined at the time of surgery. The inclusion criteria were patients who required implant surgery for adjacent missing maxillary teeth or for a totally edentulous maxilla. Given that this study is practise-based, patients were only excluded when there was not enough bone available for safe implant placement and if they were undergoing Bisphosphonate treatment or had undergone head and neck radiotherapy. Smokers were not excluded, as previously mentioned.

This research protocol was run in line with the Helsinki Declaration [23]. The Ethical Committee of Ghent University Hospital approved both the original treatment protocol and the re-evaluation [ID B67020084288]. All of the assessed patients signed an informed consent form, agreeing to the participation and use of their personal data for a scientific report, according to the requirements of the ethical committee of Ghent University Hospital.

### 3.7. Variables

The following variables were documented: patient details as well as an updated medical history; implant specifications and radiographs of the implants using a long-cone parallel technique; plaque and bleeding indices as defined by Silness–Loë [24]; a 6-point pocket chart; and complications; which were subdivided into technical, biological, and aesthetic.

### 3.8. Measurements

The software used for the radiographic analysis was AxioVision Rel. 4.8 (Carl Zeiss MicroImaging GmbH, Oberkochen, Germany), which has an accuracy of 0.01 mm. The known distance between the threads of the implant (0.66 mm) was used to calibrate the radiographs. The implant-abutment connection was used as the reference point (0 mm) from which the closest bone-to-implant contact was measured. Mesial and distal bone levels were measured. The marginal bone level was calculated by comparing the most recent peri-apical radiograph with the baseline (in this case, taken after implant insertion on the day of surgery). Bone level changes were calculated by deducting the bone level from the baseline radiograph from the bone level on the most recent radiograph. If the result was positive, this indicated bone loss or an increase in the distance between the implant-abutment connection (=reference point) and the first bone-to-implant contact, and vice versa.

The soft tissue was scored based upon the Silness-Loë plaque and bleeding index [24]. The pocket depth was measured at six points around the implant using a periodontal Williams probe. An average measurement was calculated for each implant. Peri-implantitis was defined according to the 2017 consensus report published by Berglund et al. [14], whereby the bone level is located “at least 3 mm apical to the most coronal portion of the intraosseous part of the implant, as well as a probing depth of >5 mm combined with bleeding on probing” [14].^.^

Complications were sub-divided into technical (implant fracture, screw or abutment fracture/loosening, retention loss of the prosthesis, loss of occlusal filling), aesthetic (fracture of the veneering material), and biological (peri-implantitis, abscess formation). Note was taken as to whether each complication was reversible or not, and if applicable, how the complication was resolved.

### 3.9. Bias

The analysis of the radiographs was carried out by an independent researcher not involved in the surgical or prosthetic interventions (ENN), so as to avoid a potential source of bias.

### 3.10. Study Size

The study size was predetermined by the original patient group.

### 3.11. Statistical Analysis

The analysis of the relation between type of guide and number of complications was performed by applying the Fisher Exact test to a cross table between type of guide and number of complications per patient. For the analysis of PPD, the six PPD scores per implant were averaged to arrive at one score per implant. A multilevel regression model with a random intercept for the patient was applied to study the relation between PPD as an independent variable, the type of guide as an independent variable, and implant precision (operationalised by coronal deviation and apical deviation). For bone level, a similar model was used. Here, the dependent variable was bone level at the last observation. In comparison with the PPD model, the bone level at the start was added as an extra independent variable. As unfavourable distribution of the residuals was to be expected, bootstrapping with 400 replicates was applied to both regression models. For all analyses, R version 4.1.3 was used. The multilevel analyses were performed using the lme4 library (versions 1.1-31).

## 4. Results

### 4.1. Population

Sixteen patients, out of 26, participated in the current study, of whom 8 were female and 8 were male. Of the 10 who could not attend, 3 had passed away, 3 could not be contacted due to moving addresses, 2 refused to participate, 1 could not leave home due to health problems (and has since passed away), and 1 patient continuously missed several appointments despite verbally confirming the appointments each time. The average age of the evaluated participants was 58.5 years (SD 10.8; range 27.8–73.8). The mean follow-up time was 9.1 years (SD 1.1; range 7.3–11.3). Two patients out of 16, both belonging to the mucosa group, were smokers at the time of follow-up. Eight had smoked in the past; however six of these patients had given up smoking since placement. Implant specifications are given in Table 1 and Table 2.

Results for the clinical outcomes of the patients and implants included in this study are summarized in Table 3. Two implants have failed since placement, bringing the total number of implants evaluated to 67 and a survival rate of 97.1%. Both of the failed implants belonged to patients in the mucosa-supported guide group. The specifications of the failed implants are as follows: patient 7 (active smoker), implant locus 26, length 11 mm, diameter 4.2 mm; patient 11 (former smoker), implant locus 14, length 11 mm, diameter 3.6 mm.

### 4.2. Radiographic Bone Level Changes

**Figure 1:**
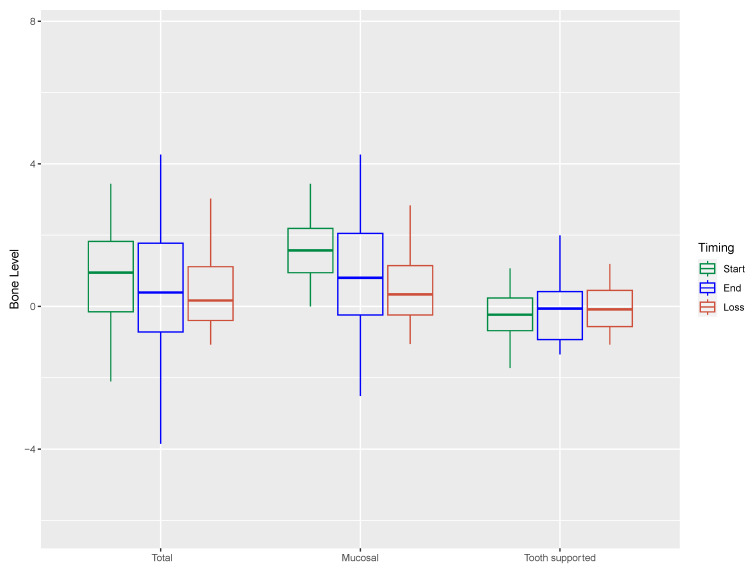
Boxplot of the median bone level in mm at baseline, at the end of follow-up, and the overall bone loss for mucosa-supported guides, tooth-supported guides, and the whole group.

Figure 1 summarizes the bone level changes—in this case, bone loss—between baseline and an average of 9.1 years of follow-up. There was no statistically significant difference between the groups (*p* = 0.27). 

**Figure 2:**
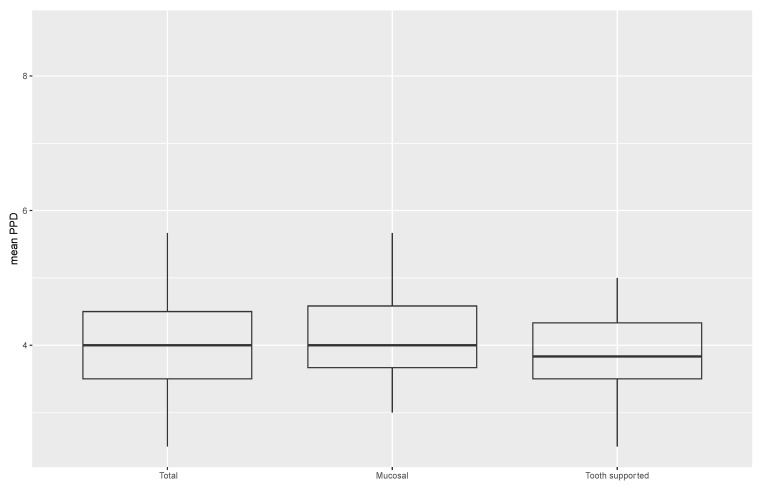
Boxplot of the median PPD in mm for the whole group, mucosa-, and tooth-supported guides.

There was, however, a statistically significant difference between groups when measuring the probing pocket depth (PPD); implants in the tooth-supported guide group yielded a lower PPD than those in the mucosa-supported group (*p* = 0.03). Figure 2 shows the effect of the guide on probing pocket depth as well as the median probing pocket depth for the whole patient group.

Table 4 shows the implant distribution divided into pocket depth and bone loss as measured at the time of implant placement. Given that 4 baseline radiographs were unreadable and two implants had failed since placement, the number of implants whereby bone loss could be measured was 63 (out of a total of 69, including the 2 failures). Based on the cross-table combining bone loss and pocket depth, it can be construed that 4 implants (6.3%) exhibited peri-implantitis, as they displayed a bone loss of >3 mm as well as a probing depth of >5 mm [14].

### 4.3. Accuracy Analysis

Coronal deviation was lightly associated with having a negative impact on the bone level at follow-up; however, this was not statistically significant (*p* = 0.167). Apical deviation did not have a statistically significant effect on bone level either. Results showing the effect of the guide as well as the accuracy of placement on bone loss are shown in Table 5.

Both coronal and apical deviations showed no statistically significant effect on probing pocket depth. The results of the effect of accuracy of placement and the type of guide are summarized in Table 6.

### 4.4. Complications

**Scheme 1:**
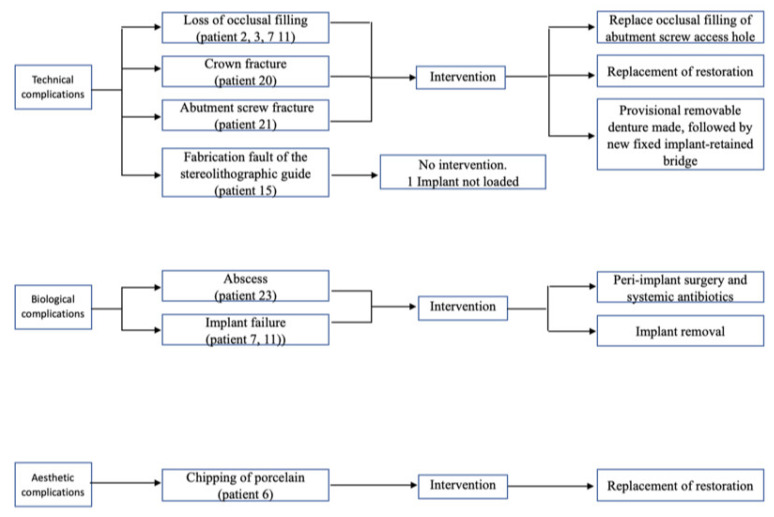
Flowchart showing details of the complications encountered, the corresponding number of patients who experienced that complication, and the subsequent intervention or treatment, if any.

Nine out of 16 patients (56%) have experienced complications since the implants were inserted. The types of complications encountered by each patient have been categorized into technical, biological, and aesthetic complications and are summarized in Table 7. Seven out of 16 patients (43.8%) experienced one or more technical complications. Biological complications were seen in 3/16 patients (18.75%), and 1/16 patients (6.25%) experienced an aesthetic complication. The Fisher’s Exact Test showed no statistically significant differences in the number of complications between groups (*p* = 1.0). The details of the complications and their subsequent intervention or treatment are shown in Scheme 1. 

All aforementioned patients who experienced a complication were followed up with an intervention, except for patient 15. In this case, a fabrication fault of the stereolithographic guide caused a mis-angulation in the placement of one of the implants. The implant in question was not loaded and thus not included in the bone level or PPD calculations.

## 5. Discussion

This observational cohort study describes the up-to 11-year clinical and radiographic outcome of maxillary implants that were placed using stereolithographic guides and flapless surgery. Our main goal was to evaluate, in the long-term, if accuracy of implant placement is positively correlated to peri-implant bone health and reduced probing pocket depth. At the time the current study started, there were no publications available reporting on this.

Out of the 26 patients originally treated as part of a cohort, 16 participated in the current follow-up, 7–11 years after implant placement. The dropout rate was 38.4% at the patient level and 41.2% at the implant level. One of the reasons for this high drop-out rate was that 4 of the patients had passed away, bringing the total down to 22 patients. If we consider this, then the drop-out rate is in fact 27% at the patient level, after a mean follow-up time of 9.1 years. Long-term studies reporting on more than 10 years of data are scarce. One study, following implants for 1–10 years, reports a drop-out rate of 7.1% on patient level [16]. This is lower than the drop-out experienced in this paper; however, it should be borne in mind that it was not in the scope of that study to follow-up the patients for such a long period. Patients were referred back to their dentists for maintenance. Another notable remark is that we are dealing with edentulous patients. They, for the most part, belong to an older age category with a lower socio-economic background. Loss to follow-up is unavoidable with time, even when implementing the best study design and conduct [25].

Older and more vulnerable patients are more likely to relocate due to a higher degree of dependency on family members, healthcare personnel or nursing facilities. This sometimes makes it quite difficult to discover their actual place of residence.

This study reports an implant survival rate of 97.1% after a mean follow-up of 9.1 years. This is comparable to the literature [10,16,26,27,28]. A recent systematic review evaluating failure rates of implants placed with guided vs. non-guided surgery found the incidence of implant failure in guided surgery to be 2.25% [29]. This demonstrates that guided implant surgery results in a high survival rate. Another author reported 100% implant survival after 10 years of clinical follow-up [30]. However, it should be noted that the study focuses on implants placed in the mandible, which could be linked to a lower failure rate than those placed in the maxilla due to the quality of the bone [31,32]. Lekholm and Zarb [33] classified bone quality by the amount of cortical and trabecular bone, ranging from a thin layer of cortical bone encompassing low-density trabecular bone (Type IV) to more dense cortical bone (Type I). In the present study, the two implant failures both belonged to the patient group treated with a mucosally supported guide; one was an active smoker, and the other was a former smoker (30 cig/day for more than 40 years). It is interesting that the paper reporting on the original patient group 1 year after placement found that the smoking status of the patient was the only significant factor (*p* = 0.001) affecting the implant survival [22]. Therefore, a statistical analysis was carried out to see if smoking still had any significant impact on the current results, and it was deduced that this was not the case, neither for bone level change nor for PPD. This could be due to the fact that six of the eight smokers in the current study quit smoking since implant surgery, thus making it statistically impossible to draw conclusions.

Our results showed that, after 7–11 years of follow-up, mean bone loss was 0.63 mm for the whole group, 0.17 mm for the tooth-supported group, and 0.91 mm for the mucosa-supported group. This is lower than the findings of one systematic review and meta-analysis evaluating implants inserted with sCAIP in healed sites, which reported a pooled marginal bone loss of 1.48 mm in 748 implants after 3-year follow-up [34], with other systematic reviews reporting similar results of 1.45 mm bone loss after 1–4 years [12], and 0.7 ± 1.3 mm after 3 years [13]. In the present study, there was no statistically significant difference between tooth-and mucosa-supported guides regarding peri-implant bone loss. However, implants with a higher coronal deviation at placement also had more bone loss at follow-up, although not statistically significant (*p* = 0.167). It sounds logical that coronal deviation would be more relevant than apical deviation when considering peri-implant bone health.

The current study reports a mean PPD of 4.24 mm for the total patient group and 3.79 mm and 4.51 mm for tooth- and mucosa-supported guides, respectively. A statistically significant difference was seen, whereby the implants in the tooth-supported guide group yielded lower PPD than those in the mucosa-supported group (*p* = 0.03). This can perhaps be attributed to the fact that the patients in the mucosa group are edentulous; there are no natural teeth neighbouring the implants. Without natural teeth present, the periodontal ligament disappears, as does the bundle bone [35]. Therefore, pocket depths tend to be higher around implants without neighbouring natural teeth. It should be noted, however, that maintaining oral hygiene around a full fixed prosthesis is more difficult compared to a partial bridge. There was no significant effect of the accuracy of implant placement on the long-term PPD. In general, the mean PPD in this study was comparable to other studies [36].

Computer-assisted implant placement is always accompanied by a substantial amount of extra cost for the patient, as well as extra time and cost investment for the clinician to correctly plan and fabricate a surgical template (planning time, stereolithographic printing, and cost of the software license). One could assume that computer-assisted implant placement may reduce the incidence of peri-implantitis by facilitating precise implant positioning and minimizing surgical trauma. This should reduce plaque accumulation around implants, potentially lowering the risk of peri-implantitis and other biological complications [11,37,38]. However, few studies report on this, and they mostly provide conflicting results. Incidence of peri-implantitis in sCAIP ranges from 14% after a follow-up time of 1–4 years [12] to only 1.7% after a 10-year follow-up period [16]. This variation between studies can be attributed to fickle definitions, numerous reporting methods, and varying study characteristics [15]. According to the 2017 consensus report [14], peri-implantitis was seen in 6.3% of implants, which is within the range found in the, albeit limited, literature. Presently, it is more or less accepted that when an adequate width of keratinised and/or attached mucosa is present around implants, it is linked to less plaque accumulation, a lower incidence of peri-implantitis, and thus fewer biological complications [39]. Although this information was not considered during surgery, as it was not in the scope of the study, it would be an interesting parameter to consider for future research.

In comparison to the literature, the complications experienced in this study were far higher than in other studies reporting rates as low as 7.1% technical and 1.7% biological complications after 10 years [16]. The types of technical and biological complications experienced were similar to those reported in other studies [9,12,12,16,17], although the majority of reviews or clinical studies report on complications experienced during implant surgery itself, such as surgical template fractures or misfits of the surgical template during surgery [10,12,40]. In the present study, one patient experienced a severe complication during surgery, whereby one implant out of four, was misplaced due to a misfabrication of the tooth-supported stereolithographic guide. This not only resulted in a higher value for global coronal and apical deviation for all 4 implants, but the misplaced implant was also unable to be loaded as part of the final prosthesis. The results from this study and other systematic reviews suggest that flapless sCAIP is not void of positioning errors [11]. It is crucial for clinicians to be aware of the potential complications, both technical and biological, even with advanced surgical techniques such as sCAIP. More research is needed to prove whether higher precision during implant placement contributes to superior results in terms of peri-implant health or complications.

A limitation of this study was the large dropout rate. As mentioned, this is a retrospective follow-up of a prospective cohort study; therefore, it was not in the scope of the initial study to follow-up the participants after this many years, and there was no treatment or control group, as one would find in a double-blind prospective clinical trial. Another drawback is that examiner calibration was not carried out to account for intra-operator variability. It is important that future research consider a larger sample size and incorporate appropriate measures to account for intra-operator variability, ensuring more robust and reliable conclusions.

## 6. Conclusions

- While computer-assisted surgery may contribute to a faster, more predictable, and precise implant placement procedure, the long-term clinical effect on survival, bone remodelling, and peri-implant health is similar to a conventional nonguided approach.
- The prevention of peri-implant disease and complications involves a multidimensional approach that includes careful patient selection, proper surgical technique, consideration of safety margins, and diligent long-term maintenance by both the patient and clinician.

## Figures and Tables

**Table 1:** Implant type divided per treatment group.

Implant Platform (mm)	Tooth-Supported	Mucosa-Supported	Total
X-Small (3.0)	0	1	1
Regular (3.5)	18	20	38
Regular (4.0)	9	18	27
Large (5.0)	0	3	3
Total	27	42	69

**Table 2:** Implant length divided per treatment group.

Implant Length (mm)	Tooth-Supported	Mucosa-Supported	Total
8	0	6	6
9	6	1	7
11	14	19	33
13	7	9	16
15	0	7	7
Total	27	42	69

**Table 3:** Overview of the total number of patients and implants, bone level measurements, PPD, BOP, and implant deviations in relation to the type of surgical guide used.

		Total Patient Group	Tooth-Supported Guide	Mucosa-Supported Guide
Number of patients	Baseline	26	13	13
	Follow-up	16	9	7 (2 implants failed in 2 patients)
Number of implants	Baseline	114 (four with unreadable baseline radiograph)	36	78
	Follow-up	67	27	40
Bone level at baseline (mm)	Mean	0.87	−0.12	1.47
	(SD; range)	(1.28; −2.11…3.44)	(0.90; −1.73…1.96)	(1.10; −2.11…3.44)
Bone level at follow-up (mm)	Mean	0.24	−0.29	0.57
	(SD; range)	(2.09; −6.10…4.26)	(1.97; −6.10…2.84)	(2.18; −5.26…4.26)
Bone loss (mm)	Mean	0.63	0.17	0.91
	(SD; range)	(1.90; −3.20…7.63)	(1.46; −3.20…4.37)	(2.09; −2.69…7.63)
PPD (mm)	Mean	4.24	3.79	4.51
	(SD; range)	(1.25; 2.50…8.67)	(0.97; 2.50…7.17)	(1.33; 3.00…8.67)
BOP (Absolute no. of implants per patient)	Mean	2.41	2.45	2.38
	(SD; range)	(0.49; 2.0…3.00)	(0.51; 2.0…3.0)	(0.50; 2.0…3.0)
Global Deviation (mm)	Mean global coronal deviation	0.85	0.82	0.87
	(SD; range)	(0.48; 0.19…2.46)	(0.50; 0.19…1.93)	(0.48; 0.29…2.46)
	Mean global apical deviation	1.14	1.13	1.14
	(SD; range)	(0.61; 0.21…3.01)	(0.68; 0.21…2.70)	(0.56; 0.32…3.01)

**Table 4:** Table showing number of implants with corresponding bone loss (mm) and probing pocket depth (mm). Four implants were classified as having peri-implantitis, as denoted in bold.

	Pocket Depth				
Bone Loss	≤3 mm	>3 mm and ≤4 mm	>4 mm and ≤5 mm	>5 mm	Total
<0 mm	2	17	6	2	27
≤1 mm	3	11	3	2	19
>1 ≤2 mm	1	1	3	2	7
>2 ≤3 mm	0	1	3	0	4
>3 mm	0	2	0	**4**	6
Total	6	32	15	10	63

**Table 5:** Table demonstrating the effect of the type of guide and the accuracy of placement on bone level at the end in mm.

	Variable	Effect	95% CI	*p*-Value
Bone Level at Follow-up (mm)	Intercept	−0.333	[−1.67…1.04]	0.648
	Bone level at baseline	0.974	[0.490…1.42]	<0.001
	Guide: Tooth- vs. mucosa-supported	0.646	[−0.518…1.89]	0.265
	Coronal Deviation	−1.12	[−2.77…0.478]	0.167
	Apical Deviation	0.383	[−1.00…1.74]	0.594

**Table 6:** Table demonstrating the effect of the type of guide and the accuracy of placement on probing pocket depth (PPD) in mm.

	Variable	Effect	95% CI	*p*-Value
PPD (mm)	Intercept	4.91	[4.03…5.75]	<0.001
	Guide: Tooth- vs. mucosa-supported	−1.02	[−1.91…−0.098]	0.030
	Coronal Deviation	−0.143	[−1.11…0.951]	0.877
	Apical Deviation	−0.224	[−0.954…0.615]	0.672

**Table 7:** Table showing distribution of all complications encountered by each patient, per treatment group, from baseline until the time of follow-up.

Type of Guide	Patient ID	Total No.	Technical	Complications Biological	Aesthetic
Tooth-supported	14				
	15	1	1		
	16				
	17				
	18				
	19				
	20	3	3		
	21	1	1		
	23	1		1	
Mucosa-supported	1				
	2	1	1		
	3	1	1		
	4				
	6	2			2
	7	2	1	1	
	11	2	1	1	

## Data Availability

The data presented in this study are available on request from the corresponding author. The data are not publicly available due to ethical restrictions.
